# FTY720 controls disease severity and attenuates sciatic nerve damage in chronic experimental autoimmune neuritis

**DOI:** 10.1186/s12974-019-1441-4

**Published:** 2019-03-02

**Authors:** Laurent Kremer, Omar Taleb, Nelly Boehm, Ayikoe Guy Mensah-Nyagan, Elisabeth Trifilieff, Jérôme de Seze, Susana Brun

**Affiliations:** 1 Biopathologie de la Myéline, Neuroprotection et Stratégies Thérapeutiques, INSERM U1119/Université de Strasbourg, Faculté de Médecine, 11 rue Humann, 67085 Strasbourg, France; 2 Fédération de Médecine Translationnelle de Strasbourg (FMTS), Strasbourg, France; 3 Department of Neurology, University Hospital of Strasbourg, Strasbourg, France; 4 Faculty of Medicine, Institute of Histology, University of Strasbourg, Strasbourg, France

**Keywords:** Chronic inflammatory demyelinating polyradiculoneuropathy, c-EAN, FTY720, Inflammatory neuropathies

## Abstract

**Background:**

Chronic inflammatory demyelinating polyradiculoneuropathy (CIDP) is an autoimmune-mediated inflammatory disease of the peripheral nervous system characterized by a response directed against certain myelin proteins and for which therapies are limited. Previous studies have suggested a beneficial role of FTY720, a sphingosine 1-phosphate (S1P) receptor agonist, known to deplete lymphocytes from the peripheral blood by sequestering them into lymph nodes, in the treatment of experimental autoimmune neuritis (EAN). Therefore, we investigated whether FTY720 is also beneficial in chronic experimental autoimmune neuritis (c-EAN), a recently developed rat model mimicking human CIDP.

**Methods:**

c-EAN was induced in Lewis rats by immunization with S-palm P0(180–199) peptide. Rats were treated with FTY720 (1 mg/kg) or vehicle intraperitoneally once daily from the onset of clinical signs for 18 days; clinical signs were assessed daily until 60 days post-immunization (dpi). Electrophysiological and histological features were examined at different time points. We also evaluated the serum levels of different pro- and anti-inflammatory cytokines by ELISA or flow cytometry at 18, 40, and 60 dpi.

**Results:**

Our data demonstrate that FTY720 decreased the severity and abolished the chronicity of the disease in c-EAN rats. Therapeutic FTY720 treatment reversed electrophysiological and histological anomalies, suggesting that myelinated fibers were subsequently preserved, it inhibited macrophage and IL-17^+^ cell infiltration in PNS, and it significantly reduced circulating pro-inflammatory cytokines.

**Conclusions:**

FTY720 treatment has beneficial effects on c-EAN, a new animal model mimicking human CIDP. We have shown that FTY720 is an effective immunomodulatory agent, improving the disease course of c-EAN, preserving the myelinated fibers, attenuating the axonal degeneration, and decreasing the number of infiltrated inflammatory cells in peripheral nerves. These data confirm the interest of testing FTY720 or molecules targeting S1P in human peripheral neuropathies.

**Electronic supplementary material:**

The online version of this article (10.1186/s12974-019-1441-4) contains supplementary material, which is available to authorized users.

## Background

Chronic inflammatory demyelinating polyradiculoneuropathy (CIDP) is considered to be an autoimmune-mediated disease affecting the peripheral nerves. It is thought to involve both cellular and humoral immunity that can be directed against specific components of the myelin sheath and/or the axon. The disease course is usually separated into chronic, progressive, or relapsing–remitting forms and can be either a sensory or a motor polyradiculoneuropathy causing symmetric, proximal, or distal muscle weakness that develops over more than 2 months. CIDP is the most common chronic autoimmune neuropathy and is pathologically characterized by focal inflammatory demyelination followed by axonal degeneration [1–5].

Currently, there are only three validated treatments for CIDP. The treatment options comprise suppression of autoimmune responses by intravenous immunoglobulins, plasma exchange, or corticosteroids, which have proven efficacy and are used in the short and long term. However, some difficulties need to be considered: even if about two thirds of patients respond to at least one of these three therapies, a small percentage of patients do not respond to any therapy; the serious long-term side effects of corticosteroids; and treatment availability, cost and safety, and adverse events related to administration routes [4, 6–12]. Therefore, there is a need to establish more effective therapeutic strategies for chronic inflammatory polyneuropathies.

We recently developed a new model of chronic experimental autoimmune neuritis (c-EAN) that can be easily and reliably induced by active immunization of Lewis rats with the P0(180–199) peptide thiopalmitoylated (S-palm P0(180–199)) at cysteine 181 [13, 14], with selective involvement of the peripheral nervous system (PNS). In contrast to the classical acute monophasic EAN induced by P0(180–199) and mimicking Guillain-Barré syndrome, 100% of rats immunized with S-palmP0(180–199) develop an ongoing neuropathy, either chronic or relapsing, that fulfills electrophysiological criteria of demyelination with axonal degeneration, confirmed by immunohistopathology. Interestingly, the late phase of the chronic disease is characterized by accumulation of interleukin-17^+^ (IL-17^+^) cells, macrophages, and T cells in sciatic nerves and by high IL-17 levels in the serum. The c-EAN model bears considerable similarity to CIDP [1], supporting the use of this model for translational drug studies.

FTY720 (fingolimod) is a synthetic analog of sphingosine 1 phosphate (S1P), which has a high bind affinity to S1P receptors, particularly to S1P_1_ and S1P_3–5_ [15, 16]. Indeed, FTY720 undergoes phosphorylation in vivo by sphingosine kinase 2 [17] for converting to its active form, FTY720-phosphate. This form acts through S1P receptor present in various human and rodent organ systems including immune, vascular, and nervous systems (e.g., astrocytes in the central nervous system and dorsal root ganglia and Schwann cells in PNS) [18–24], thereby regulating a variety of processes. The better known is the immunological process, whereby FTY720 prevents the egress of lymphocytes from secondary lymphoid organs resulting in a reduced lymphoid cell count in peripheral blood [16, 25–27]. Due to its effect to deplete peripheral lymphocytes, FTY720 provides therapeutic benefits in animal models of multiple sclerosis and in patients with relapsing–remitting multiple sclerosis by reducing the infiltration of lymphocytes into the central nervous system, thereby reducing neuroinflammation [28–30]. In three phase 3 clinical trials, oral FTY720 significantly reduced patients’ relapse rate and MRI lesion activity and decreased the risk of disability progression when compared to the placebo group (TRANSFORMS, FREEDOMS I, FREEDOMS II) [31–33]. As a result, in 2010, FTY720 became the first oral immunomodulator to be approved by the Food and Drug Administration followed by the European Medicines Agency in 2011 for relapsing–remitting multiple sclerosis treatment. However, FTY720 has some side effects that have also been found in animal models: the most common are the risk of cardiac conduction disorders (bradycardia, atrioventricular block) and mild hypertension requiring cardiovascular monitoring at first administration, elevation of liver enzymes requiring regular biological monitoring, and a risk of macular edema detected by systematic ophthalmological examination [34–38].

The therapeutic effect of FTY720 has also been highlighted in EAN animal models. It was shown to be an effective immunomodulatory agent by ameliorating the disease course, decreasing the number of infiltrating macrophages and T cells and inhibiting the accumulation of IL-17^+^ cells in peripheral nerves of EAN rats [39, 40]. The prophylactic effect of orally administered FTY720 was studied in EAN rats and demonstrated an at least indirect neuroprotective effect in the PNS (reduced expression of myeloid precursor protein and reduced Schwann cell apoptosis) [41]. The beneficial therapeutic effect of orally administered FTY720 was also studied in a mouse model of spontaneous autoimmune polyneuropathy [42]. The efficacy, safety, and tolerability of oral FTY720 have already been tested in the FORCIDP randomized controlled trial, but lack of efficacy led to study discontinuation [43]. However, several methodological concerns may be raised regarding this protocol, especially the delay for the efficacy of FTY720. Because several hypotheses could explain these results, we decided to investigate the effect of therapeutically administered FTY720 in our c-EAN rat model, which shares many of the clinical, electrophysiological, and histological features of human CIDP.

## Methods

### Peptide synthesis

P0(180–199) peptide [ACKRGRQTPVLYAMLDHSRS], S-palmitoylated P0(180–199) peptide [AC(palm)KRGRQTPVLYAMLDHSRS], obtained by thiopalmitoylation of residue cysteine at position 181, was synthesized in our laboratory as previously described [13].

### Animals

Male Lewis rats (Charles River, L’Arbresle, France), 7–8 weeks old and weighing 210–230 g were used in the present study. All experiments were approved by the animal experimentation ethics committee of the University of Strasbourg, France.

### Induction of disease and assessment of clinical scores

The procedures were as previously described [14, 44]. Rats were anesthetized intraperitoneally with ketamine chlorhydrate (37 μg/g)/xylazine (Rompun) (5.5 μg/g). To induce c-EAN, rats were immunized with S-palm P0(180–199), by subcutaneous injection at the base of the tail of 200 μL of an emulsion containing 200 μg of peptide and 0.5 mg of *Mycobacterium tuberculosis* (strain H37 RA, Difco, Detroit, Michigan, USA) emulsified in 100-μL saline and 100-μL incomplete Freund’s adjuvant (IFA) (Sigma-Aldrich, St. Quentin Fallavier, France). For comparison, the classical acute EAN model was induced in Lewis rats by immunization with P0(180–199) peptide and rats immunized with complete Freund’s adjuvant (CFA) alone were used as negative controls. Body weight and clinical score were assessed daily from day 0 until 60 days post-immunization (dpi). Severity of paresis was graded as follows: 0, no illness; 1, flaccid tail; 2, moderate paraparesis; 3, severe paraparesis; 4, tetraparesis; 5, death; and intermediate scores of 0.5 increments were given to rats with intermediate signs.

### Treatment with FTY720

FTY720 (Cayman Chemical, Ann Arbor, Michigan, USA) was dissolved in EtOH/saline (1:10). c-EAN rats were treated with freshly prepared FTY720 or with vehicle (EtOH/saline [1:10]). One milligram per kilogram FTY720 in 1 ml EtOH/saline (1:10) (vehicle) was intraperitoneally injected once daily from 2 days after disease onset until 31 dpi. This dosage was applied because it has been shown to ameliorate EAN in rats [39].

### Electrophysiological studies

Electrophysiological studies were performed at 13, 18, 40, and 60 dpi in treated and non-treated animals using the same method as already described for the characterization of the c-EAN model [14]. Briefly, rats were anesthetized as described above, and the sensory nerve action potential (SNAP) was recorded on the caudal nerve using two needle electrodes (ALPINE-bioMed, Natus France) inserted at the base of the tail, with the stimulating electrodes placed about 50 mm distally. Sciatic nerve motor conduction was assessed by examining the amplitude and the latency of the evoked compound muscle action potentials (CMAP). A stimulating needle electrode was inserted at the hip of the animal (proximal) or at the knee (distal) and two recording needle electrodes (Alpine-bioMed, Natus France) were inserted in the gastrocnemius muscle. Recordings were obtained on a differential amplifier DAM8 (WPI, London, UK) with a filter setting of 10 Hz to 10 kHz. The stimulus waveform (square shape stimuli, 0.1 ms duration) was generated with Clampex acquisition software (P-clamp8 software package, Axon Instruments, LA, US) and the signal was acquired through a Digidata 1224 (Axon Instruments, LA, USA).

### Histological studies

To evaluate pathological changes and inflammatory cell infiltration in the PNS, control rats and FTY720- or vehicle-treated rats were sacrificed at 18 and 60 dpi. Rats were deeply anesthetized with ketamine/xylazine; proximal sections of sciatic nerves (four rats/group) were then collected and fixed overnight with 2.5% of glutaraldehyde in cacodylate buffer, post-fixed for 2 h in 1% osmium tetroxide, embedded in epon 812 resin, and polymerized at 60 °C for 48 h. Semi-thin transverse sections of 0.5-μm thickness were cut on an ultramicrotome, stained with toluidine blue, and observed by light microscopy (Olympus BX60) connected to a DP7V digital camera. The myelinated fiber density (number of myelinated fibers/mm^2^) was performed in two regions of interest of 100 × 100 μm each one per slide. For the percentage of small and large myelinated fibers, 300 myelinated fibers per sciatic nerve were randomly analyzed and divided in two groups according to their normal size distribution in control sciatic nerve (i.e., small fibers with a perimeter < 19 μm and large fibers with a perimeter > 19 μm; Additional file 1: Figure S1). Counting was performed by one observer blind to the therapy received, using ImageJ software. For immunohistochemistry, sciatic nerves and spinal nerve roots (five rats in control and vehicle-treated group and ten rats in FTY720-treated group) were also dissected out, fixed in Bouin-Hollande solution, embedded in paraffin, and serially sectioned (5-μm-thick sections). After dewaxing, cross sections were heated at 80 °C for 10 min in citrate buffer. Endogenous peroxidase was inhibited with 0.02% H_2_O_2_ in water for 10 min. Non-specific binding sites were blocked with 5% fetal calf serum (Gibco Invitrogen, Camarillo, CA, USA) in phosphate-buffered saline (PBS) for 30 min and then sections were incubated overnight with the following monoclonal antibodies: anti-myelin basic protein (anti-MBP) (1:200; produced in house) for myelin; anti-neurofilaments (1:1000; clone SMI-31; Abcam) for phosphorylated neurofilaments H; anti-CD68 (1:400; clone ED1; Serotec, Oxford, UK) for macrophages; and anti-IL-17 (1:100; Santa Cruz Biotechnology, Santa Cruz, CA, USA). Antibody binding to tissue sections was visualized with biotinylated anti-mouse immunoglobulin G (IgG; 1:200; Vectastain®, Vector Laboratories, Burlingame, CA, USA) and avidin–biotin complex (ABC-peroxidase kit; Vectastain®, Vector Laboratories), followed by development with 3,3′-diaminobenzidine (DAB) substrate (Vector® DAB SK-4100, Vector Laboratories) for IL-17 and VIP substrate (Vector® VIP SK-4600, Vector Laboratories) for other antibodies. Slides were viewed using a Nikon Eclipse E600® optical microscope connected to a Nikon® Digital Sight DS-Fi1 digital camera. Images were processed using Nikon NIS Elements® software supplied with the camera. Macrophage and IL-17^+^ cell counting was performed on five cross sections per rat in a region of interest of 330 × 430 μm per slide. Counting was performed by two observers blind to the therapy received, using ImageJ software. Results are given as mean values per square millimeter.

### Cytokine secretion

Sera from control, FTY720-, or vehicle-treated rats were collected at 18, 40, and 60 dpi. The concentration of IL-17 cytokine was measured in duplicate in undiluted sera using commercial ELISA kits specific for rat IL-17 (eBioscience, San Diego, CA, USA), as per the manufacturer’s instructions. Results were expressed as pg/mL relative to a standard curve (limit for positivity 2 pg/mL). The concentration of interferon gamma (IFNγ), tumor necrosis factor (TNF), IL-4, and IL-10 was measured in undiluted sera using a cytometric bead array kit (Rat Flex Set, BD Pharmingen, San Jose, CA, USA) according to the manufacturer’s instructions. Cytokine concentrations were presented as picograms per milliliter relative to a standard curve. The theoretical limits of detection were 6.8 pg/mL IFNγ, 27.7 pg/mL TNF, 3.4 pg/mL IL-4, and 19.4 pg/mL IL-10.

### Antibody measurements

Sera from FTY720- or vehicle-treated rats were also tested at 18, 40, and 60 dpi for the presence of anti-P0(180–199) antibodies using ELISA. P0(180–199) peptide was coated onto 96-well plates at 20 μg/mL in 0.05 M carbonate-bicarbonate buffer solution (pH 9.6, 100 μL/well) and incubated overnight at 4 °C. Plates were then washed with PBS and blocked with 1% bovine serum albumin in PBS for 1 h at 37 °C. After washing, sera (100 μL/well) diluted at 1/5000 were added in duplicate and incubated for 2 h at 37 °C. After washing, plates were incubated with goat anti-rat IgG coupled to peroxidase (1:2000, SIGMA-Aldrich) for 2 h at 37 °C. After extensive washing, each well was incubated with 75 μL of 3,3′,5,5′-tetramethylbenzidine at room temperature until color development. The reaction was stopped by addition of 1 M H_2_SO_4_ (25 μL/well). Results were expressed in optical density (OD) at 450 nm.

### Statistical analysis

Results are presented as mean ± standard error of the mean (SEM). Data were analyzed using GraphPad Prism 6 (GraphPad Software Inc., San Diego, CA, USA). Statistical analyses were determined by ANOVA followed by the Bonferroni correction method (or Kruskal-Wallis test for nonparametric data). Significance levels were set at *p* < 0.05.

## Results

### Therapeutic FTY720 treatment decreases disease severity in c-EAN rats and abolishes the chronicity of the pathology

**Fig. 1:**
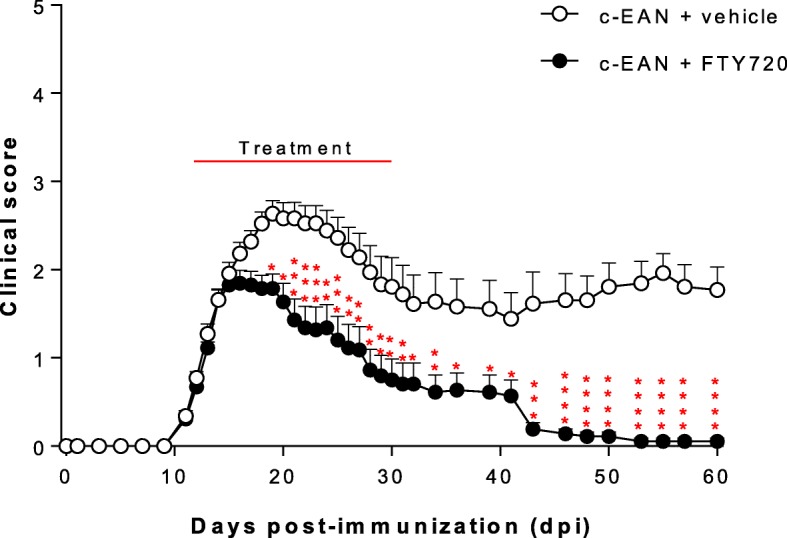
Effect of FTY720 on the clinical course of c-EAN. Clinical score values were measured in vehicle- (white circle) and FTY720-treated (black circle) rats. Therapeutic injections of FTY720 or vehicle were administrated intraperitoneally from 12 to 30 days post-immunization (dpi). Mean values, SEM, and *p* values are indicated. *p < 0.05; **p < 0.01; ***p < 0.001; ****p < 0.0001. For vehicle-treated group, *n* = 22 from 0 to 18 days post-immunization (dpi), *n* = 18 from 19 to 40 dpi, and *n* = 13 from 41 to 60 dpi; for FTY720-treated group, *n* = 26 from 0 to 18 dpi, *n* = 22 from 19 to 40 dpi, and *n* = 18 from 41 to 60 dpi. *n*, number of rats

To examine the effect of FTY720 on c-EAN rats, animals were intraperitoneally treated with FYT720 (1 mg/kg) once daily for 18 days from day 12 post-immunization. All c-EAN rats treated with vehicle (EtOH/saline; 1:10) developed a chronic type of disease with an onset at 10.5 ± 0.5 dpi, a maximal clinical score of 2.7 ± 0.1 at 18 dpi without recovery, and were still exhibiting clinical signs at 60 dpi. In contrast, FTY720 therapeutic treatment not only decreased the maximal clinical scores of the chronic-EAN rats compared to vehicle treatment (1.8 ± 0.2 at 16 dpi) but also abolished the chronicity of the disease. Differences between the vehicle and FTY720 groups were statistically significant from 18 up to 60 dpi (Fig. 1). The classical acute EAN model, induced with P0(180–199), was used for comparison. FTY720 treatment decreased the maximal clinical scores compared to vehicle treatment and allowed faster recovery in the EAN rats (Additional file 2: Figure S2). Similar results have already been demonstrated by Zhang et al. [39].

The evolution of body weight during the disease course is shown in Additional file 3: Figure S3. A maximal weight loss that corresponded to the maximal clinical scores of the disease and was followed by weight gain was observed in c-EAN (A) and EAN (B) rats treated with vehicle. FTY720 did not attenuate the loss of body weight in c-EAN rats. In contrast, in the classical acute EAN group, FTY720 treatment induced an apparent, but smaller weight loss at maximal clinical score, but the difference in weight change failed to reach statistical significance.

### Beneficial effect of FTY720 treatment on electrophysiological changes

**Fig. 2:**
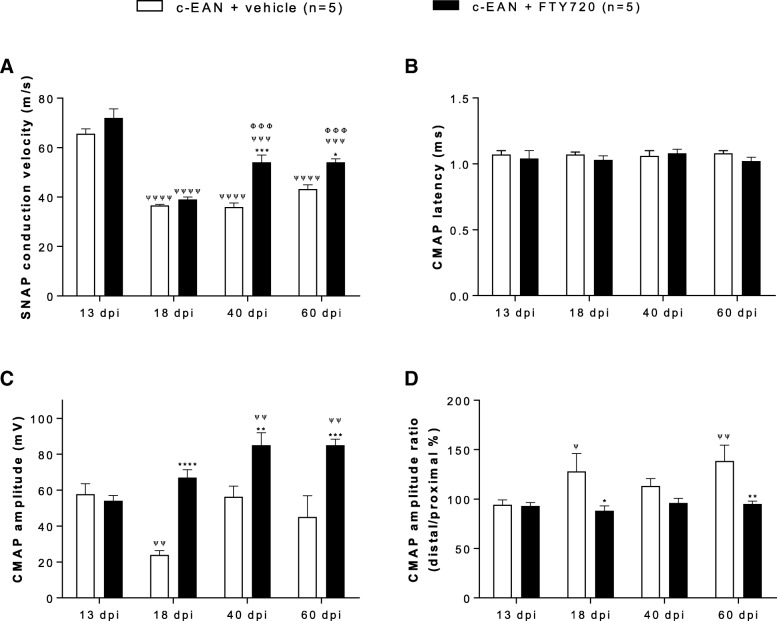
Electrophysiological changes in c-EAN rats treated with FTY720 or vehicle at 13, 18, 40, and 60 dpi. **a** Sensory nerve action potential (SNAP) conduction velocity of the caudal nerve. **b** Sciatic motor nerve distal CMAP latency. **c** CMAP amplitudes obtained after proximal stimulation of the sciatic nerve. **d** CMAP amplitude ratio of distal values/proximal values. Mean values, SEM, and *p* values are indicated. *p values refer to difference between FTY720-treated group and the vehicle-treated group at the same dpi (*p < 0.05; **p < 0.01; ***p < 0.001; ****p < 0.0001). ^ψ^*p* values refer to the difference in the vehicle- or FTY720-treated group when compared to 13 dpi (^ψ^*p* < 0.05; ^ψψ^*p* < 0.01; ^ψψψ^*p* < 0.001; ^ψψψψ^*p* < 0.0001). ^Φ^*p* values refer to the difference in the FTY720-treated group when compared to 18 dpi (^ΦΦΦ^*p* < 0.001). *n* = 5 rats/group. *n*, number of rats

To verify the potential therapeutic effects of FTY720 on demyelination and axonal degeneration, we performed electrophysiological measurements on five c-EAN rats treated with vehicle and five c-EAN rats treated with FTY720 at 13 dpi (onset of the clinical signs of the disease), at 18 dpi (maximal clinical scores of the disease), at 40 dpi (the middle of the disease), and at 60 dpi (the late chronic phase of the disease). As shown in Fig. 2a, the sensory nerve conduction velocity (SNCV) for c-EAN rats treated with vehicle was significantly and strongly decreased at 18 dpi (37 ± 0.4 m/s) compared to 13 dpi (66 ± 2 m/s) and no recovery was observed at 40 or 60 dpi (36 ± 1.7 m/s and 43 ± 1.8 m/s). In contrast, although a significant reduction of SNCV was also observed at 18 dpi for the c-EAN rats treated with FTY720 (39 ± 1 m/s) compared to 13 dpi (72 ± 3.7 m/s), this was followed by a middle recovery at 40 and 60 dpi (55 ± 3 m/s and 55 ± 1.4 m/s, respectively) that was not significant compared to 13 dpi but significantly increased compared to 18 dpi. These results suggest that a demyelination of sensory fibers persisted in c-EAN rats treated with vehicle and that this was restored following FTY720 treatment.

CMAP latencies and amplitudes were also measured at 13, 18, 40, and 60 dpi. CMAP latency in c-EAN rats treated with vehicle or FTY720 did not differ significantly from 13 dpi at all other time points (Fig. 2b). Maximal CMAP amplitudes in the c-EAN group treated with vehicle were significantly and strongly reduced at 18 dpi (23.9 ± 2.4 mV) and were increased at 40 and 60 dpi (56.2 ± 5.9 mV and 45.1 ± 11.8 mV, respectively) when compared to 13 dpi (57.7 ± 5.8 mV). These data are indicative of an axonal degeneration/regeneration. In contrast, CMAP amplitude in the group treated with FTY720 was increased at 18 and 40 dpi (67 ± 4.4 mV and 85 ± 6.9 mV, respectively) and was maintained at 60 dpi (85 ± 3.3 mV), when compared to the vehicle-treated group (24 ± 2.4 mV, 56 ± 5.9 mV and 45 ± 11.8 mV, respectively) (Fig. 2c). When compared to 13 dpi (54 ± 3.1 mV), the CMAP amplitude in the group treated with FTY720 was increased only at 40 and 60 dpi. These results are indicative of axonal regeneration.

The distal and proximal CMAP maximal amplitudes were also measured at 13, 18, 40, and 60 dpi. In the group treated with vehicle, the maximal CMAP amplitude distal/proximal ratio was 37% higher at 18 dpi and 47% higher at 60 dpi (128 ± 18 mV and 138.5 ± 16 mV, respectively) compared to 13 dpi (94 ± 5 mV), suggesting the presence of a conduction block mechanism between the two sites of stimulation at the hip and knee. No significant changes were observed in the CMAP amplitude distal/proximal ratio for the group treated with FTY720 when compared to 13 dpi (Fig. 2d).

### FTY720 preserves myelinated fibers and attenuates axonal loss in sciatic nerves of c-EAN rats

**Fig. 3:**
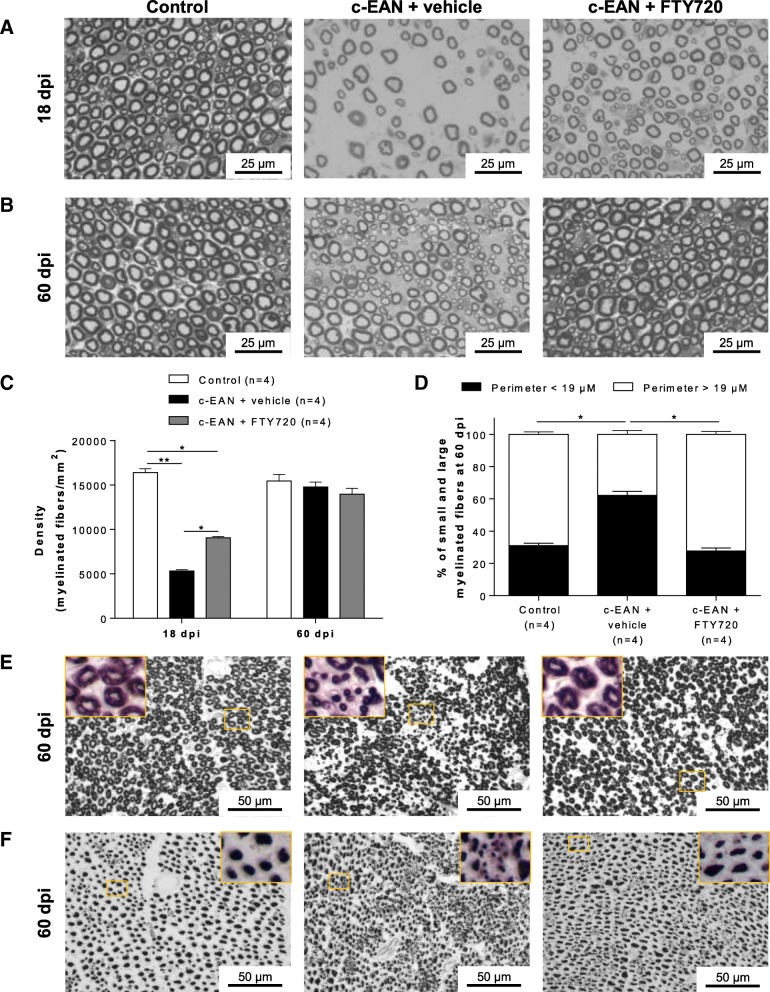
FTY720 attenuates the severity of alterations occurring in sciatic nerves of c-EAN rats. Representative semi-thin cross-sections of sciatic nerves from control and c-EAN rats treated with FTY720 or vehicle (three groups of rats) and stained with toluidine blue at **a** 18 dpi and **b** 60 dpi. **c** Number of myelinated fibers per square millimeter at 18 and 60 dpi and **d** percentage of small (perimeter < 19 μm) and large (perimeter > 19 μm) myelinated fibers at 60 dpi in sciatic nerves from control and c-EAN rats treated with FTY720 or vehicle. Mean values, SEM, and *p* values are indicated. *p < 0.05; **p < 0.01. *n*, number of rats. Representative paraffin cross sections of sciatic nerves taken from the three groups of rats and labeled with **e** anti-MBP antibody for myelin and **f** anti-SMI-31 antibody for phosphorylated neurofilament at 60 dpi

To evaluate if therapeutic FTY720 treatment induced morphological changes of myelinated fibers in sciatic nerves of c-EAN rats, histological studies were carried out on sciatic nerves from control (CFA-immunized rats), vehicle-, and FTY720-treated c-EAN rats at 18 and 60 dpi. Semi-thin transverse sections of sciatic nerves demonstrated that clinical disability of c-EAN rats was associated with marked reduction of myelinated fibers and axonal degeneration at 18 dpi followed by regeneration along with the presence of numerous thinly myelinated axons at 60 dpi when compared to sciatic nerves from the control group, which presented a well-defined structure and uniform myelin sheath thickness (Fig. 3a–d and Additional file 1: Figure S1). Treatment of c-EAN rats with FTY720 preserved myelinated fibers and reduced the extent axonal degeneration at 18 dpi and restored myelinated fibers to a normal appearance at 60 dpi.

These results were confirmed by immunohistochemical staining of myelin and neurofilaments of sciatic nerves at 60 dpi (Fig. 3e, f). Vehicle-treated c-EAN rats were characterized by a reduction in the number of large-diameter myelinated fibers and the presence of numerous clustered axons of small diameter and very few axons of normal diameter compared to the normal appearance of sciatic nerves from the control group. FTY720 treatment resulted in the presence of myelinated fibers of comparable size and appearance to those of control animals.

### FTY720 decreases the number of infiltrated inflammatory cells in the PNS of c-EAN rats

**Fig. 4:**
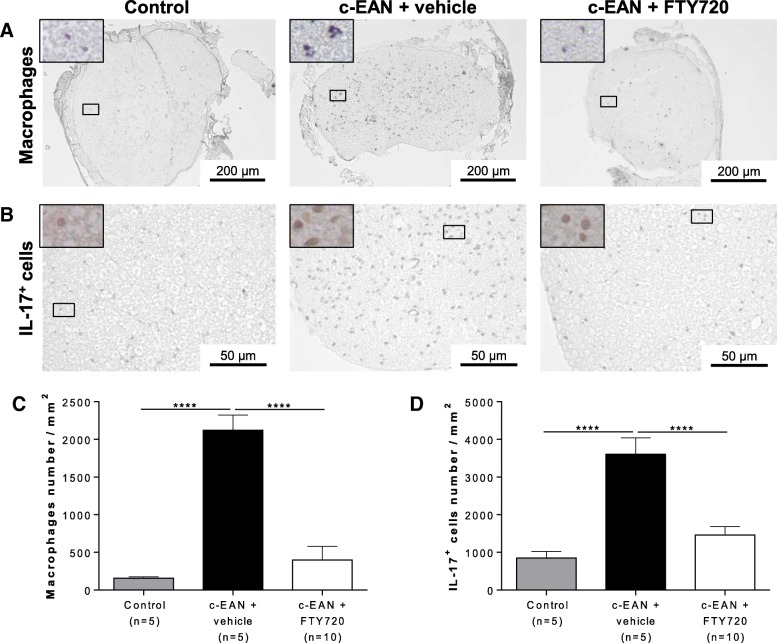
Therapeutic FTY720 treatment inhibits inflammatory cell (macrophages and IL-17^+^ cells) infiltration in sciatic nerves of c-EAN rats. **a** Representative cross sections of sciatic nerves from control and c-EAN rats treated with FTY720 or vehicle (three groups of rats) were labeled with anti-CD68 antibody for macrophage infiltration at 60 dpi. **b** Representative cross sections of sciatic nerves sampled in the three groups of rats were labeled with anti-IL-17 antibody for IL-17^+^ cell infiltration at 60 dpi. **c** Macrophage and **d** IL-17^+^ cell numbers per square millimeter at 60 dpi in sciatic nerves from control and c-EAN rats treated with FTY720 or vehicle. Mean values, SEM, and *p* values are indicated. ****p < 0.001. *n*, number of rats

The infiltration of macrophages was assessed at 60 dpi in sciatic nerves from control, vehicle-, or FTY720-treated c-EAN rats at 60 dpi (Fig. 4a, c). Sciatic nerves from vehicle-treated c-EAN rats were characterized by an accumulation of macrophages when compared to control rats. Therapeutic FTY720 treatment of c-EAN animals resulted in a significant decrease in the number of infiltrated macrophages in sciatic nerves when compared to vehicle-treated animals.

Th17 cells, producing the cytokine IL-17, have been shown to play an important role in the pathology of various autoimmune diseases. We therefore stained for IL-17-producing cells in the spinal nerve roots of vehicle- or FTY720-treated c-EAN rats at 60 dpi (Fig. 4b, d). The chronic late phase of the disease (60 dpi) was characterized by an accumulation of IL-17^+^ cells in vehicle-treated c-EAN rats but not in control animals. Treatment of c-EAN rats with FTY720 significantly decreased the accumulation of infiltrated IL-17^+^ cells in spinal nerve roots when compared to vehicle treatment.

Macrophage or IL-17 cell infiltration in classical EAN rats treated with vehicle or FTY720 at 60 dpi did not differ significantly from control rats (data not shown).

### Therapeutic FTY720 treatment reduces the serum levels of IL-17, IFNγ, and TNF pro-inflammatory cytokines in c-EAN rats

**Fig. 5:**
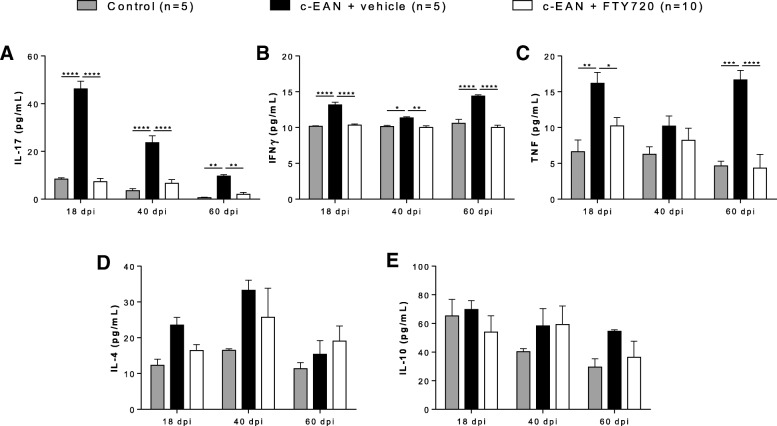
Effect of FTY720 on serum IL-17, IFNγ, TNF, IL-4, and IL-10 cytokine. Cytokine levels were evaluated at different stages of the disease (18, 40, and 60 dpi) in the serum collected from control rats that received CFA alone or from c-EAN rats that were treated with FTY720 or vehicle. **a** IL-17 level was evaluated by ELISA. **b**–**e** IFNγ, TNF, IL-4, and IL-10 cytokine levels were measured using cytometric bead array technique. Mean values, SEM, and *p* values are indicated. *p < 0.05; **p < 0.01; ***p < 0.001; ****p < 0.0001. *n*, number of rats

We also examined whether the benefits of FTY720 treatment in reducing inflammatory cell infiltration in PNS and improving clinical symptoms of c-EAN were accompanied by a reduced level of peripheral pro- and anti-inflammatory cytokines. IL-17, IFNγ, TNF, IL-4, and IL-10 levels were measured in the sera of control, vehicle-, or FTY720-treated c-EAN rats collected at 18, 40, and 60 dpi. FTY720 treatment significantly reduced IL-17 and IFNγ pro-inflammatory cytokine levels in the c-EAN group at any stage of the disease, whereas TNF level was only reduced at 18 and 60 dpi. There were no significant differences for circulating IL-4 and IL-10 anti-inflammatory cytokines among the three groups (Fig. 5).

### FTY720 decreases the levels of antibody responses against P0(180–199) antigenic peptide

**Fig. 6:**
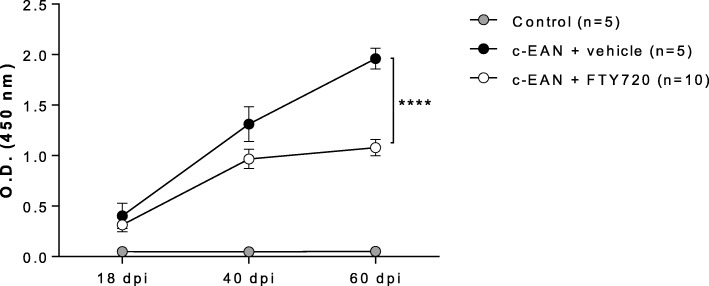
Effect of FTY720 on IgG specific antibody response. IgG antibody response to P0(180–199) was evaluated at different stages of the disease (18, 40, and 60 dpi) in the serum collected from control rats that received CFA alone or from c-EAN rats that were treated with FTY720 or vehicle. Mean values, SEM, and *p* values are indicated. ****p < 0.0001. *n*, number of rats

The ability of S-palm P0(180–199) peptide to induce P0(180–199)-specific IgG antibody responses was investigated at 18, 40, and 60 dpi by measuring the level of these antibodies circulating in the serum of control, vehicle-, and FTY720-treated c-EAN rats. A strong IgG antibody response to P0(180–199) was measured in vehicle-treated c-EAN rats (readily detectable in the samples collected at 18 dpi and increasing with time). FTY720 treatment significantly reduced the antibody reactivity at 60 dpi (Fig. 6). Antibody response to P0(180–199) peptide was hardly detectable in the control group.

## Discussion

There are currently very few validated treatments for human CIDP, the most common chronic autoimmune neuropathy, which has a prevalence ranging from one to nine cases per 100,000 individuals [45–47]. Although the majority of patients can be successfully treated by intravenous immunoglobulin therapy, plasma exchange, or corticosteroids, a large number of patients with CIDP are non-responders or become refractory to these treatments, and some are left with permanent disability [1, 4, 45, 48–52]. Furthermore, these treatments are only transitorily effective and patients relapse when the drug is stopped. Novel therapeutic options, offering high efficacy and fewer secondary side effects, are thus eagerly awaited. To investigate potential new therapeutic strategies, the use of reliable and easily reproducible animal models is essential. In this study, we investigated the therapeutic effects of FTY720, a S1P receptor agonist known to deplete lymphocytes from peripheral blood by sequestering them in lymph nodes, in c-EAN, a rat model mimicking CIDP, which has proved useful in investigating new therapeutic strategies [53]. The results of our experiments showed a beneficial effect of FTY720 in our c-EAN rat model. FTY720 administered therapeutically (1 mg/kg) by intraperitoneal injections not only decreased the severity of c-EAN but also abolished the chronicity of the disease. Similar results have been reported in the literature in a mouse model developing spontaneous autoimmune polyneuropathy (SAP) but with FTY720 administered orally (same dose as in our study) [42]. Another study in the SAP model has since suggested otherwise but these contradictory results could be explained by the fact that, in the latter study, treatment began when demyelination had most likely further progressed [54].

Furthermore, therapeutic FTY720 treatment in c-EAN animals resulted in the attenuation of axonal degeneration and the preservation of myelinated fibers. FTY720 also reduced macrophage infiltration and accumulation of IL-17^+^ cells in PNS at the late phase of the chronic disease. Interestingly, FTY720 reduced the levels of circulating IL-17, IFNγ, and TNF pro-inflammatory cytokines and the levels of antibody responses against P0(180–199) antigenic peptide. These results are consistent with the most robust mechanism of action of FTY720, to sequester lymphocytes in lymph nodes and block their trafficking to the target organs [27, 55], thereby inducing a reduction in critical pro-inflammatory mediators in the blood, including IL-17, IFNγ, and TNF. Indeed, additional mechanisms have been attributed to the effects of FTY720. In vitro studies have demonstrated the implication of S1P in Th17 cell development and have suggested that FTY720 immunosuppression could be partially attributable to Th17-mediated inflammation [56]. The immunopathogenesis of CIDP remains poorly understood, but it has recently been suggested that Th17 may be an important determinant in the evolution of CIDP [57]. It was also shown that IL-17^+^ cells accumulation in EAN sciatic nerves temporally correlated with the severity of the neurological signs [40]. Our results are consistent with the possible involvement of IL-17^+^ cells in the chronicity of the disease. Interestingly, the therapeutic activity of FTY720 in the c-EAN model could be monitored by measurement of circulating IL-17 and anti-P0(180–199) antibodies, which could therefore be used as biological markers.

The efficacy and safety of 0.5 mg FTY720 administered orally once daily versus placebo in 106 patients with CIDP was recently the subject of a clinical study (FORCIDP) [43]. Unfortunately, the study had to be discontinued due to lack of efficacy. Among the possible explanations for these results, it was suggested that several aspects of the study design were not appropriate to show the efficacy of the drug, especially the delay for FTY720 efficacy [58]. Indeed, in the FORCIDP trial, it was evaluated a short-term management of a recently stabilized disease (60% of participants in both groups, placebo or FTY720, were free from worsening in the 6 months before screening) that was then destabilized by an abrupt stopping of usual therapy (this was even more true in the subgroup of participants who had previously received intravenous immunoglobulin), based on the hypothesis that FTY720 is a fast-acting drug. However, it has been suggested that FTY720 is, relative to at least natalizumab [59], slow to act in multiple sclerosis [30]. It has also been suggested that pretreatment procedure is essential to define risk and benefit for each patient previously to FTY720 start [60]. The use of FTY720 in our animal model shows results that contradict those of FORCIDP trial in humans. This can be explained by experimental modalities which were not the same: the delay of follow-up which was probably shorter in our model, not allowing to eliminate a relapse on longer term; the dose of FTY720 used in our model was slightly higher than the dose used in humans; there could be probably physiopathological differences between our model and human CIDP. Our results and current studies suggest that other synthetic analogs of S1P and more selective S1P receptor modulators that have or may have therapeutic effects similar to those of FTY720 could be tested in immune-mediated peripheral neuropathies such as CIDP, provided that care is taken with the choice of patients and previous treatment, selecting the appropriate inclusion criteria and the appropriate primary endpoint.

## Conclusion

The experimental data we generated showed FTY720 treatment to be an effective immunomodulatory agent that improved the disease course in c-EAN, preserved myelinated fibers, and decreased the axonal degeneration. More specifically, FTY720 reduced the accumulation of IL-17^+^ cells and infiltrating macrophages in PNS and abnormally elevated soluble pro-inflammatory IL-17, IFNγ, and TNF cytokine levels circulating in the serum, factors that characterize c-EAN rats in the late phase of the chronic disease. These findings show that FTY720 displays remarkable beneficial effects both at the biological and clinical levels. Even if FTY720 was evaluated in a clinical trial in patients with CIDP and has proven limited effects compared to placebo [43], showing its efficacy in vivo in our rat model provides evidence that FTY720 and its analogs could be tested in immune-mediated peripheral neuropathies. It also highlights the potential of molecules targeting S1P for innovative drug development in autoimmune-mediated demyelinating neuropathies.

## Additional files

Additional file 1: **Figure S1.** Distribution of myelinated fibers perimeter at 60 days post-immunization (dpi) in sciatic nerve from control and c-EAN rats treated with FTY720 or vehicle. Mean values and SEM are indicated. *n*, number of rats. (PDF 11 kb)

Additional file 2: **Figure S2.** Effect of FTY720 on the clinical course of EAN. Clinical score values were measured in vehicle (white square) and FTY720-treated (black square) rats. Therapeutic injections of FTY720 or vehicle were administrated intraperitoneally from 12 to 30 days post-immunization (dpi). Mean values, SEM and *p* values are indicated. *p < 0.05; **p < 0.01; ***p < 0.001; ****p < 0.0001. *n*, number of rats. (PDF 14 kb)

Additional file 3: **Figure S3.** Effect of FTY720 on the body weight of c-EAN and EAN rats. Clinical score values were measured in vehicle (white circle, white square) and FTY720-treated (black circle, black square) rats. Therapeutic injections of FTY720 or vehicle were administrated intraperitoneally from 12 to 30 days post-immunization (dpi). Mean values and SEM are indicated. (PDF 17 kb)

## Abbreviations

c-EAN: Chronic experimental autoimmune neuritis
CFA: Complete Freund’s adjuvant
CIDP: Chronic inflammatory demyelinating polyradiculoneuropathy
CMAP: Compound muscle action potential
dpi: Days post-immunization
IFNγ: Interferon gamma
IgG: Immunoglobulin G
IL: Interleukin
MBP: Myelin basic protein
P0: Myelin protein zero
PNS: Peripheral nervous system
S1P: Sphingosine 1 phosphate
SNAP: Sensory nerve action potential
S-palm: Thiopalmitoylated
TNF: Tumor necrosis factor
