# Supplementary figures and images for: FTY720 controls disease severity and attenuates sciatic nerve damage in chronic experimental autoimmune neuritis
Source: J Neuroinflammation. 2019 Mar 2;16:54. doi: 10.1186/s12974-019-1441-4 (PMC6397476; doi:10.1186/s12974-019-1441-4)

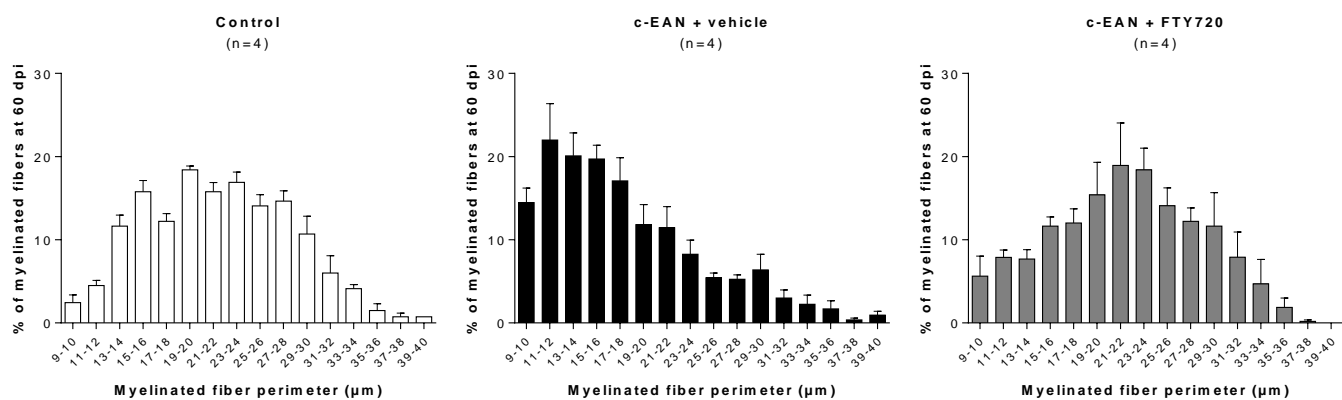

**Additional file 1: Figure S1**

Supplement: Supplementary file 1 — Figure S1. Distribution of myelinated fibers perimeter at 60 days post-immunization (dpi) in sciatic nerve from control and c-EAN rats treated with FTY720 or vehicle. Mean values and SEM are indicated. n, number of rats. (PDF 11 kb) [file 12974_2019_1441_MOESM1_ESM.pdf]

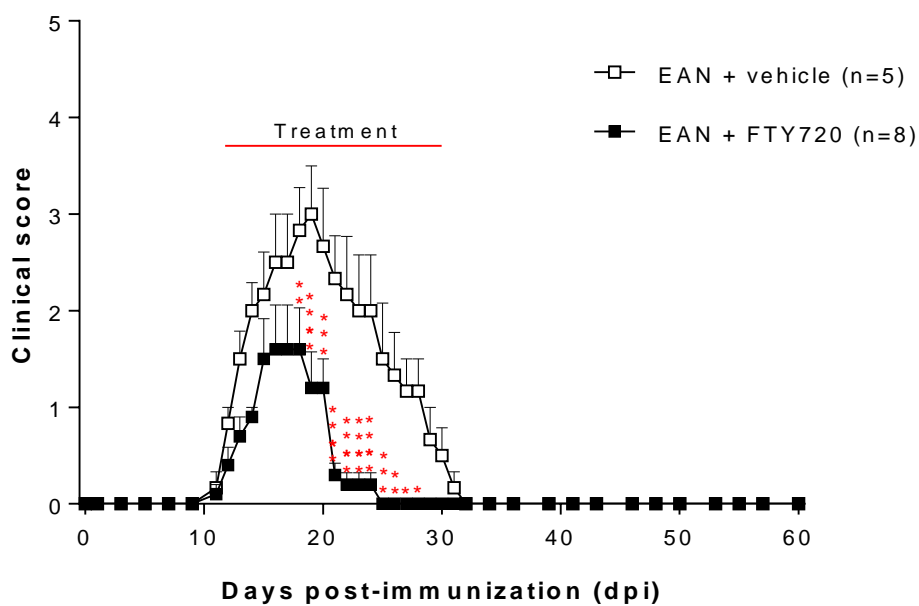

**Additional file 2: Figure S2**

Supplement: Supplementary file 2 — Figure S2. Effect of FTY720 on the clinical course of EAN. Clinical score values were measured in vehicle (white square) and FTY720-treated (black square) rats. Therapeutic injections of FTY720 or vehicle were administrated intraperitoneally from 12 to 30 days post-immunization (dpi). Mean values, SEM and p values are indicated. *p < 0.05; **p < 0.01; ***p < 0.001; ****p < 0.0001. n, number of rats. (PDF 14 kb) [file 12974_2019_1441_MOESM2_ESM.pdf]

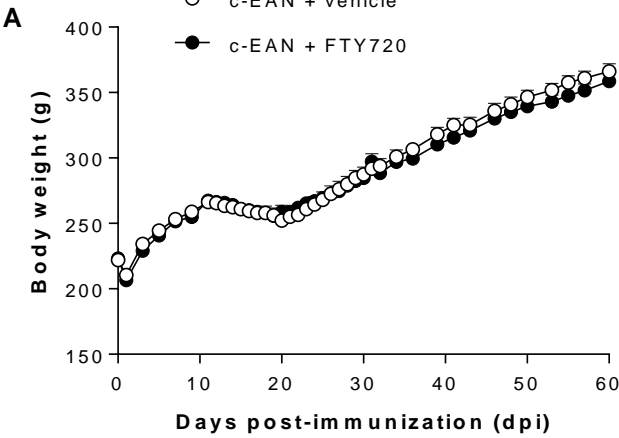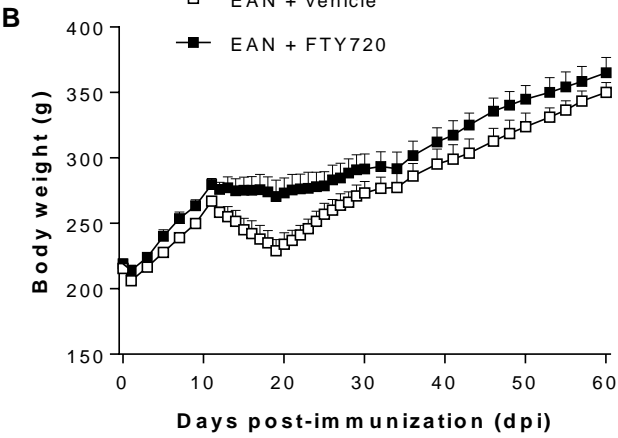

Additional file 3: Figure S3

Supplement: Supplementary file 3 — Figure S3. Effect of FTY720 on the body weight of c-EAN and EAN rats. Clinical score values were measured in vehicle (white circle, white square) and FTY720-treated (black circle, black square) rats. Therapeutic injections of FTY720 or vehicle were administrated intraperitoneally from 12 to 30 days post-immunization (dpi). Mean values and SEM are indicated. (PDF 17 kb) [file 12974_2019_1441_MOESM3_ESM.pdf]
